# KDM6B enhances anti-PD-L1 immunotherapy efficacy by increasing CD8^+^ T-cell infiltration in colorectal cancer

**DOI:** 10.7150/jca.118571

**Published:** 2025-07-28

**Authors:** Jing Xun, Zehan Liu, Bin Liu, Xiaolin Jiang, Huichao Yang, Jinjin Liu, Botao Wang, Ruifang Gao, Aimin Zhang, Xueliang Wu, Qing Xi, Xiangyang Yu, Qi Zhang

**Affiliations:** 1 Tianjin Nankai Hospital, Tianjin Medical University, Tianjin 300100, China.; 2 Institute of Integrative Medicine for Acute Abdominal Diseases, Tianjin 300100, China.; 3 Tianjin Key Laboratory of Acute Abdomen Disease Associated Organ Injury and ITCWM Repair, Tianjin 300100, China.; 4 Department of General Surgery, The Third People's Hospital of Chengdu, Affiliated Hospital of Southwest Jiaotong University & The Second Affiliated Hospital of Chengdu, Chongqing Medical University, Chengdu, China.; 5 Department of Oncology, Chongqing Traditional Chinese Medicine Hospital, Chongqing, China.; 6 Tianjin Institute of Medical and Pharmaceutical Sciences, Tianjin 300020, China.; 7 The First Affiliated Hospital of Hebei North University, Hebei 075000, China.; 8 Department of Clinical Laboratory, National Center for Respiratory Medicine, National Clinical Research Center for Respiratory Disease, State Key Laboratory of Respiratory Disease, Guangzhou Institute of Respiratory Health, The First Affiliated Hospital of Guangzhou Medical University, Guangzhou, 510120, China.

**Keywords:** colorectal cancer, lysine-specific demethylase 6B (KDM6B), tumor immunotherapy, CD8^+^ T cells, anti-PD-L1

## Abstract

**Background:** Cytotoxic CD8^+^ T cells play a vital role in its antitumor response, and increasing their infiltration in tumors is an effective strategy for enhancing the efficacy of antitumor immunotherapy. Lysine-specific demethylase 6B (KDM6B) plays a suppressive or oncogenic role in colorectal cancer (CRC). However, the its contribution in CRC immunity remains unclear.

**Methods:** The expression of KDM6B was analyzed in CRC using TCGA database and clinical specimens. Its role in tumor growth and metastasis was assessed through in vitro assays and in vivo models, including subcutaneous xenografts and orthotopic liver metastasis mice. CD8^+^ T cell infiltration, recruitment, and activation were assessed via immunohistochemistry, transwell assay, and flow cytometry. The expression of PD-L1 and CD8^+^ T cell-recruiting chemokines CCL5, CXCL9, CXCL10 were detected. GSEA identified KDM6B-regulated signaling pathways. ChIP‒qPCR was used to determined the enrichment level of H3K27me3 on PD-L1, CCL5, CXCL9, and CXCL10 promoters. Finally, the synergistic effect of KDM6B inducer paricalcitol with anti-PD-L1 therapy was investigated using a subcutaneous xenograft mouse model.

**Results:** KDM6B was downregulated in CRC tissues and cells. KDM6B overexpression suppressed CRC proliferation, tumor growth and liver metastasis, while enhancing CD8^+^ T cells infiltration, recruitment, and functional activation. KDM6B upregulated PD-L1 and CCL5, CXCL9, CXCL1 expression in CRC cells. Mechanically, GSEA revealed JAK/STAT pathway enrichment. KDM6B increased p-STAT3 levels and the STAT3 signaling mediated the promoting effect of KDM6B on PD-L1 and chemokines expression. In addition, KDM6B overexpression reduced H3K27me3 enrichment on promoters of PD-L1 and chemokines. Finally, the combination of paricalcitol and anti-PD-L1 antibody enhanced anti-tumor efficacy in CRC mouse model.

**Conclusion:** KDM6B potentially suppresses CRC progression by enhancing CD8^+^ T cell infiltration via dual mechanisms: STAT3-mediated transcriptional activation and H3K27me3 demethylase-dependent epigenetic remodeling of PD-L1 and chemokine genes (CCL5/CXCL9/CXCL10). The synergistic effect of KDM6B inducer paricalcitol with anti-PD-L1 enhances antitumor immunity, supporting its potential combination strategy for CRC treatment.

## 1. Introduction

Colorectal cancer (CRC) has become the third most commonly diagnosed cancer according to global cancer statistics^1^. With the improvement of people's living standards and the changes in diet and lifestyle, the incidence of colorectal cancer is on the rise globally^2^. Currently, conventional treatments for CRC include surgery, chemotherapy and radiotherapy. Furthermore, these treatments can be used in combination depending on the location and progression of the disease^3^. Recent evidence reveals that tsRNA-GlyGCC promotes CRC progression and confers 5-fluorouracil (5-FU) resistance^4^; the complex signal regulatory network plays a key role in CRC progression^5^, suggesting that exploring the pathogenesis of CRC is of great significance for effective treatment of CRC.

Immunotherapy, especially immune checkpoint blockade (ICB), has become a powerful strategy for treating cancer. Although the application of immune checkpoint inhibitors has achieved significant efficacy in the treatment of melanoma and non-small cell lung cancer, the response rate of these agents in CRC patients is limited^6^. Therefore, enhancing the sensitivity and responsiveness of CRC patients to anti-PD-1/PD-L1 antibody therapy is urgent.

Studies have shown that tumor microenvironment affects tumor responsiveness to anti-PD-1/PD-L1 antibody therapy. The expression of PD-L1 and the number and activity of infiltrated CD8^+^ T cells in the tumor are two important indicators^7^,^8^. Cytotoxic CD8^+^ T cells, which are the most powerful effector cells in the antitumor immune response and the backbone of tumor immunotherapy, are recruited at the tumor site by locally secreted chemokines^8^. Increased levels of the Th1 chemokines C-C motif chemokine ligand 5 (CCL5), CXC-chemokine ligand 9 (CXCL9) and CXCL10 are associated with increased numbers of tumor-infiltrating CD8^+^ T cells^9^. However, PD-L1 exerts immunosuppressive effects through engagement of its receptor PD-1 on immune cells, triggering T lymphocyte apoptosis, functional anergy, and terminal exhaustion—thereby enabling immune evasion of PD-L1-expressing tumor cells from CD8^+^ cytotoxic T lymphocyte-mediated killing^10^,^11^. Therefore, promoting the expression and secretion of Th1 chemokines in tumor cells is significant for remodeling the tumor immune microenvironment, improving the sensitivity and effectiveness of tumor treatment with anti-PD-1/PD-L1 antibodies, and inhibiting tumor progression.

Lysine-specific demethylase 6B (KDM6B), also known as jumonji domain-containing protein D3 (JMJD3), is a demethylase that reduces H3K27me3 levels in gene promoters to promote gene expression. Many studies have demonstrated that KDM6B is positively or negatively associated with tumor progression^12^. However, the role of KDM6B in CRC is still controversial. On the one hand, KDM6B is expressed at low levels in CRC clinical samples, and low KDM6B expression is associated with poor prognosis in surgically resected CRC patients^13^,^14^. On the other hand, KDM6B was found to mediate the promoting effect of epithelial cell adhesion molecule (EpCAM) on tumor proliferation in CRC^15^. However, the role of KDM6B in CRC and its involvement in immune regulation remain poorly investigated. Therefore, this study aimed to explore the correlation between KDM6B expression and CRC progression, and further investigate the association between KDM6B and immunotherapy responses in CRC.

In this study, we found that KDM6B was downregulated in CRC tissues and cells. Overexpression of KDM6B significantly suppressed the malignant phenotypes of CRC, including cell proliferation, migration, invasion, tumor growth and liver metastasis. Furthermore, we found that KDM6B overexpression increased the infiltration of CD8^+^ T cells in mouse CRC tissues. Mechanistically, KDM6B promoted CD8^+^ T-cell infiltration by upregulating PD-L1 and the CD8^+^ T-cell-attracting chemokines CCL5, CXCL9, and CXCL10 expression, via its demethylase activity and activation of the STAT3 signaling pathway. Finally, given the previous report that paricalcitol induces KDM6B expression in CRC and breast cancer cells, we investigated the combination of paricalcitol and an anti-PD-L1 antibody for CRC therapy. Our findings showed that paricalcitol potentiated the antitumor efficacy of the PD-L1 antibody against CRC. Collectively, this study provides a theoretical foundation and a potential strategy for enhancing the efficacy of CRC immunotherapy.

## 2. Materials and Methods

### Tumor specimens

The colorectal cancer tissues and corresponding paracancerous tissues were obtained from a total of 7 patients diagnosed with CRC at Tianjin Nankai Hospital. This study was approved by the Clinical Trial Ethics Committee of Tianjin Nankai Hospital (Approval No: NKYY_YX_IRB_2018_039_01), and informed consent was obtained from all the patients involved. All tissues were immediately frozen in liquid nitrogen at the time of surgery and stored at -80 °C for further study.

### Cell lines

The human CRC cell line NCM460, HCT116, HT29, RKO, HCT15 and the murine CRC cell lines CT26, C26 and MC38 were obtained from the cell bank of the Chinese Academy of Science (Shanghai, China). The cells were cultured in high-glucose Dulbecco's modified Eagle's medium (DMEM; Invitrogen, Carlsbad, CA, USA) supplemented with 10% fetal bovine serum (Biological Industries). Murine cytotoxic T cells CTLL-2 were obtained from Wuhan Pricella Biotechnology Co., Ltd and were cultured in RPMI-1640 medium (Invitrogen) supplemented with 10% FBS, 1.0 μg/ mL concanavalin A (Biosharp, Beijing, China) and 100 U/mL recombinant murine IL-2 (Peprotech, Germany). The cells were maintained at 37 °C in a humidified atmosphere of 5% CO_2_.

### Vector construction and establishment of stable cell lines

The vector construction and establishment of stable cell lines were performed as previously described^16^. Briefly, the DNA sequence of murine or human KDM6B was amplified and cloned and inserted into the plasmid pLV-EF1α-MCS-IRES-Bsd (Addgene, Cambridge, MA). Lentiviruses carrying overexpression or empty vector (MCS) were produced as instructed. Stable recombinant cell lines (CT26-MCS, CT26-KDM6B, HCT116-MCS, HCT116-KDM6B) were established by adding lentivirus-containing medium to cells in the presence of polybrene for 48 hours, followed by selection with blasticidin.

### Real-time quantitative PCR (RT-qPCR)

Total RNA was extracted from tissues and cells by using TRIzol (Invitrogen) reagent, and reverse transcription was performed using the TransScript First-Strand cDNA Synthesis SuperMix Kit (TransGen Biotech, Beijing, China) according to the manufacturer's instructions. RT‒qPCR was performed using an ABI 7500 Real-Time PCR System (Applied Biosystems Thermo Fisher). The qPCR procedures were run using Hieff® qPCR SYBR Green Master Mix reagent (Yeasen Biotech) according to the manufacturer's instructions. The mRNA levels were normalized to those of GAPDH and were calculated by using the 2^-△△Ct^ method. Primer sequences were shown in Table 1.

### Western blot

RIPA lysis buffer (Sigma, St. Louis, MO, USA) containing protease and phosphatase inhibitors was used to obtain the protein from the cells and tissues. The protein concentrations were quantified by the Pierce™ BCA Protein Assay (Thermo Scientific, USA). Western blotting analysis were performed according to published protocols. Briefly, protein samples were separated by 10 % SDS‒PAGE and transferred to PVDF membranes. The membrane was blocked with 5 % skim milk for 1 h at room temperature and then incubated overnight with different primary antibodies at 4 ℃. The primary antibodies were used as follows: KDM6B (ab38113, Abcam), β-actin (AC026, Abclonal, Wuhan, China), Cyclin D1 (ET1601-31, HUABIO, Hangzhou, China), E-cadherin (A1149, ABclonal), N-cadherin (13116, CST), PD-L1 (A11273, ABclonal), pSTAT3 (9145, CST), STAT3 (4904, CST). After that, membranes were incubated with horseradish peroxidase-conjugated secondary antibodies for 1 h at room temperature. Then immunoreactive bands were visualized using an enhanced chemiluminescence (ECL) kit (Millipore).

### Transwell migration assay

Serum-free cell suspension was added to the Transwell chamber (8 μm pore size, 24-well plate), and 500 μL of medium containing 10% FBS was added to the bottom chamber. After culturing for 24 h, the nonmigrating cells in the upper chamber were removed by using cotton swabs, and the inserts were fixed with 4% formaldehyde for 20 min at room temperature. The migrated cells were stained with 0.1% crystal violet staining solution and visualized using an Olympus microscope (Olympus Co., Tokyo, Japan).

### Wound healing assay

The cells were seeded in a 6-well plate at a density of 1×10^6^ cells/well in DMEM containing 10% FBS. After the cells were cultured for 24 h, a pipette tip was used to scratch the cells vertically to create a “wound”. After that, the floating cells were removed with PBS, and the remaining cells were cultured with DMEM containing 2% FBS. The process of wound healing at 0 h, 24 h and 48 h was assessed by using an Olympus microscope (Olympus Co., Tokyo, Japan). The wound healing areas were quantified with ImageJ software.

### Cell viability assay

A total of 3×10^3^ cells in 100 µL of DMEM were seeded in each well of 96-well plates. Ten microlitres of CCK8 solution (Beyotime, Shanghai, China) was added to each well at 24 h, 48 h, 72 h and 96 h. After the cells were incubated for 2 h at 37 °C, the optical density (OD) was read at a wavelength of 450 nm by using a microplate reader.

### ELISA

CRC cells (MC38) were pretreated with paricalcitol (10 nM) for 24 h, and then cocultured with murine cytotoxic T cells (CTLL-2) at a ratio of 1:1 with or without an anti-PD-L1 antibody (200 nM) for 24 h. The levels of IFN-γ in the supernatants were quantified using an ELISA kit (TW8439, TONGWEI, Shanghai) according to the manufacturer's protocol.

### T-cell migration and activation assays

Human peripheral blood mononuclear cells (PBMCs) samples from healthy individuals were purchased from the Tianjin Blood Center, which was approved by the Clinical Trial Ethics Committee of Tianjin Nankai Hospital (Approval No: NKYY_YX_IRB_2018_039_01), PBMCs separation was performed by density gradient centrifugation using Ficoll-Paque (Solarbio, Beijing, China). Briefly, fresh peripheral blood was diluted with 1×PBS (Solarbio, Beijing, China), gently overlaid on Ficoll-Paque, and then centrifuged at 350×g for 30 min. After centrifugation, the middle layer was harvested, washed and resuspended with 1×PBS. Then, CD3^+^ T cells were obtained from human PBMCs by positive selection magnetic bead cell sorting (Miltenyi Biotec) of CD3^+^ lymphocytes. CD8^+^ T cells were stimulated with CD3/CD28 antibody-coated beads (Miltenyi Biotec). The migration of CD8^+^ T cells was evaluated by using a Transwell Permeable system with a 5 μm polycarbonate membrane (Costar). Activated CD8^+^ T cells (1 ×10^5^) were plated in the upper chamber. Conditioned media from HCT116 cells overexpressing KDM6B or from vector control (MCS) were added to the bottom chamber to stimulate CD8^+^ T-cell migration. The number of migrated CD8^+^ T cells was measured using a flow cytometer (Dakewe, Beijing, China).

To examine the activity of CD8^+^ T cells, the migrated cells were washed twice with cell staining buffer (CSB), and incubated with Fc block (564219, BD) for 10 minutes at room temperature. Then, the cells were incubated with anti-CD8-PE (E-AB-F1271D, Elabscience, Wuhan, China) for 30 minutes at room temperature, and washed twice with CSB. Subsequently, the cells were fixed with 3.2% paraformaldehyde for 30 minutes and permeabilized with 0.2% Triton X-100 for 10 minutes at 4℃. Finally, the cells were stained with anti-IFN-γ-APC (E-AB-F1196E, Elabscience) for 45 minutes at RT. IFN-γ^+^ CD8^+^ T cells were acquired using a flow cytometer (Dakewe).

### Animal study

Female BALB/c, BALB/c nude, and C57BL/6J mice (6-8 weeks of age) were purchased from SPF (Beijing) Biotechnology Co., Ltd. (Beijing, China) and maintained in a specific-pathogen-free facility at Nankai Hospital. A tumor xenograft or allograft mouse model was constructed by injecting HCT116 cells or CT26 cells (1 × 10^6^) expressing vector control (MCS) or KDM6B into the dorsal flanks of mice. Tumor volume was measured and calculated using the following standard formula: length× width^2^/2. After the mice were sacrificed, the tumor tissues were stored for further analysis. To establish a mouse model of liver metastasis, HCT116-overexpressing KDM6B or MCS cells (3×10^6^ cells/mouse) were injected into the spleens of the mice, and liver tissues were obtained after 4 weeks. All *in vivo* procedures were performed in accordance with protocols approved by the Animal Ethics Committee of Tianjin Nankai Hospital (NKYY-DWLL-2024-005).

For the combination therapy experiment, C57BL/6J mice were injected with 5 × 10^5^ MC38 cells into the dorsal flanks to establish a subcutaneous tumor model. Seven days later, according to the the previous research, paricalcitol (0.3 μg/kg)^16^, the anti-PD-L1 antibody (100 μg/mouse) or their combination was injected intraperitoneally every 2 days. A total of 7 injections were given.

### Hematoxylin and eosin (H&E) and immunohistochemical (IHC) staining

Tumor and liver tissues were fixed in 4% paraformaldehyde for 24 hours, embedded in paraffin, sectioned and stained with hematoxylin and eosin. IHC-stained sections were analyzed using an SP Kit (Ovitalin-Biotin Detection System for Streptomyces Rabbits) purchased from ZSGB-BIO according to the manufacturer's protocol. Brifly, paraffin-embedded tissue sections were deparaffinized using xylene and alcohol and then pre-treated with antigen retrieval in citrate buffer (pH6.0, Solarbio Science & Technology, Beijing, China). After that, sections were incubated with 3 % H_2_O_2_ and followed by blocking with 5 % goat serum. The sections were then incubated with primary antibodies at 4 ℃ overnight followed by incubation with a biotinylated secondary antibody and then incubation with an avidin-peroxidase complex. The positive signals of immunocomplexes were detected using diaminobenzidine, and the cells were counterstained with hematoxylin. Images were obtained using an inverted microscope (Olympus Co., Tokyo, Japan). The signal density of the tissue areas was measured in at least three sections.

### Flow cytometry

Cells were collected, resuspended in 1×PBS containing 1% BSA and incubated with antibodies for 30 min at room temperature in the dark. The following antibodies were obtained from Elabscience Biotechnology Co., Ltd. (Wuhan, China): APC anti-mouse CD45 (E-AB-F1136E), FITC anti-mouse CD3 (E-AB-F1013C), PE anti-mouse CD8 (E-AB-F1104D), PE anti-human PD-L1 (29E.2A3), and PE Anti-Mouse PD-L1 (10F.9G2). The cells were analyzed using an EXFLOW flow cytometer (Dakewe), and the data were analyzed using FlowJo software (Tree Star, Inc., Ashland, OR).

### Chromatin immunoprecipitation (ChIP)

The ChIP assay was performed using an EZ-ChIP kit (Cat#17-371; Millipore Corp., Billerica, MA) following the manufacturer's instructions. In brief, HCT116 cells overexpressing KDM6B or MCS cells grown in 10 cm dishes were cross-linked with 1% formaldehyde for 10 min at room temperature. The reaction was stopped with glycine. Samples were collected, sonicated, and centrifuged, and the supernatant was subsequently collected for immunoprecipitation using an antibody against H3K27me3 (ab6002, Abcam). Anti-RNA polymerase and normal mouse IgG (ab190475, Abcam) were used as positive and negative controls, respectively. Semiquantitative RT‒PCR was performed to detect DNA fragments in the gene promoters. The sequences of the gene promoters used were shown in Table 2.

### Bioinformatics analysis

The datasets for this study were obtained from public databases, including The Cancer Genome Atlas (TCGA) (https://cancergenome.nih.gov) and the Tumor Immune Estimation Resource (TIMER) database (https://cistrome.org/TIMER/). GSEA software was used to analyze the enrichment of JAK/STAT3 signaling among patients with different expression levels of KDM6B in the TCGA cohort. The TIMER database of immune cells that infiltrate tumors was used to investigate immune cell infiltration into tumors. The TIMER database contains TCGA data used to calculate gene expression associations between cancer and immune infiltration.

### Ethics approval statement

The colorectal cancer tissues and corresponding paracancerous tissues were obtained from Tianjin Nankai Hospital. This study was approved by the Clinical Trial Ethics Committee of Tianjin Nankai Hospital (Approval No: NKYY_YX_IRB_2018_039_01), and informed consent was obtained from all the patients involved. All animal experiments were performed strictly according to the guidelines for laboratory animals of Tianjin Nankai Hospital and approved by the Institutional Ethics Committees of Tianjin Nankai Hospital (Approval No. NKYY-DWLL-2024-005).

### Statistical analysis

The data were analyzed using GraphPad Prism 8 software, and the results are presented as the mean ± SEM. Student's t test or nonparametric test, two-way repeated-measures ANOVA were used to evaluate statistical significance; *P<0.05, **P<0.01, and ***P<0.001 indicate a significant difference.

## 3. Results

### KDM6B is downregulated in CRC tissues and cells

**Figure 1:**
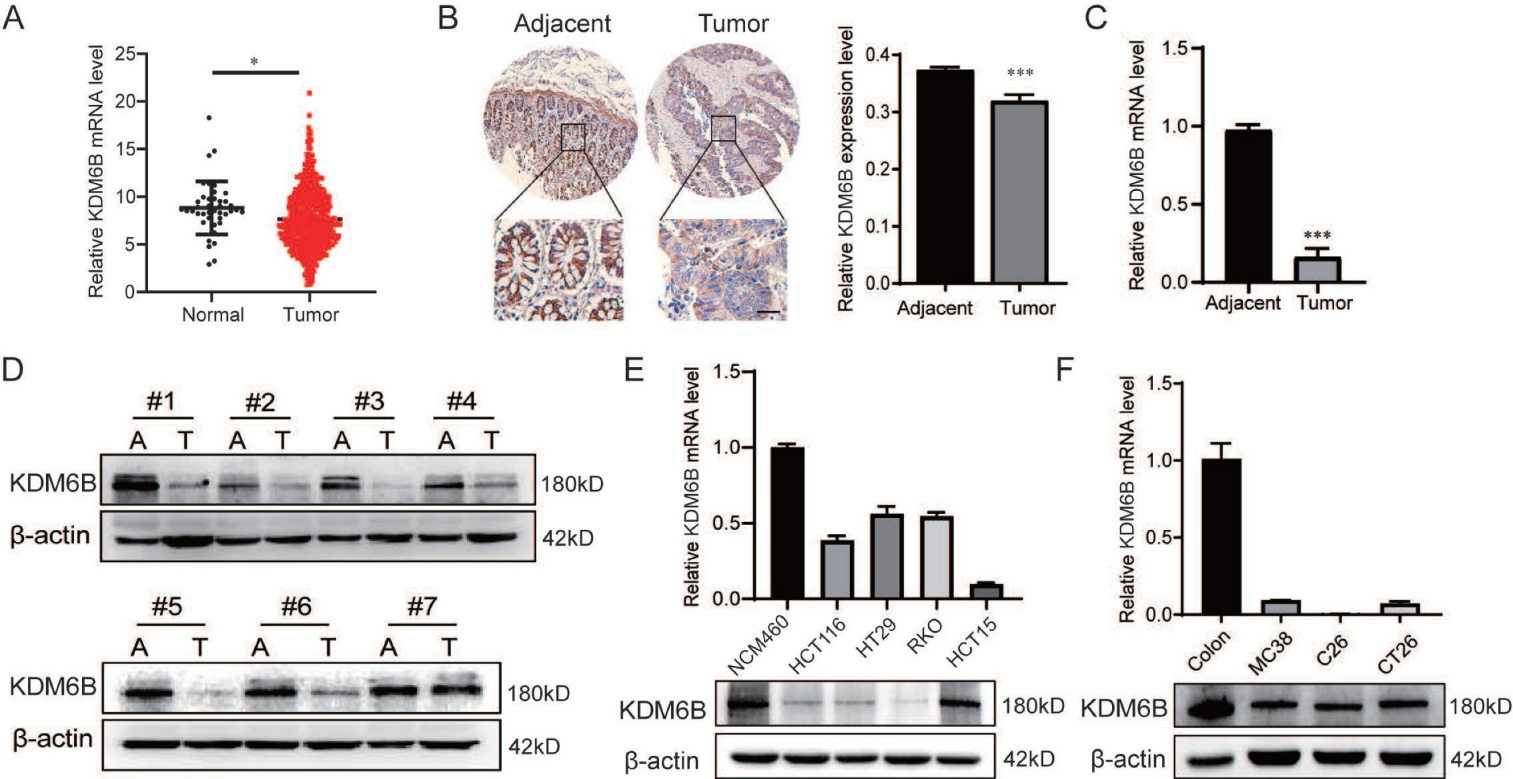
** KDM6B is downregulated in CRC tissues and cells. (A)** The TCGA database was used to analyze the expression of KDM6B in normal (n=41) and CRC tissues (n=473). **(B)** Relative KDM6B expression levels in adjacent and cancer tissue microarray (n=13) were detected via immunohistochemical (IHC) staining. **(C-D)** RT‒qPCR and Western blot analysis of KDM6B expression in CRC tumor (T) and adjacent (A) tissues. **(E-F)** RT‒qPCR and Western blot analysis of KDM6B expression in normal and CRC cells from humans (normal epithelial cells: NCM460; CRC cells: HCT116, HT29, RKO, HCT15) (E) and mice (normal colon; CRC cells: MC38, C26, CT26) (F). The data are shown as the means ± SEMs of three independent experiments. *P < 0.05, **P < 0.01, ***P < 0.001.

First, we evaluated the expression of KDM6B in CRC tissues by using a database, and the results demonstrated that the expression of KDM6B was downregulated in CRC tissues compared to that in adjacent tissues (**Fig. 1A**). In addition, we confirmed that the expression of KDM6B was lower in CRC tissues than in paracancerous tissues from microarray (**Fig. 1B**). To further confirm the above results, we examined the mRNA and protein expression of KDM6B in 7 pairs of samples from CRC patients, including adjacent and cancer tissues. The data also showed similar results (**Fig. 1C-D**). Furthermore, we explored the expression of KDM6B in various CRC cells from humans and mice. The results showed that the mRNA and protein expression of KDM6B was also lower in CRC cells than in colon cells (**Fig. 1E-F**). Taken together, these results suggest that KDM6B is downregulated in CRC tissues and cells.

### KDM6B inhibits the malignant characteristics of CRC cells *in vitro* and* in vivo*

**Figure 2:**
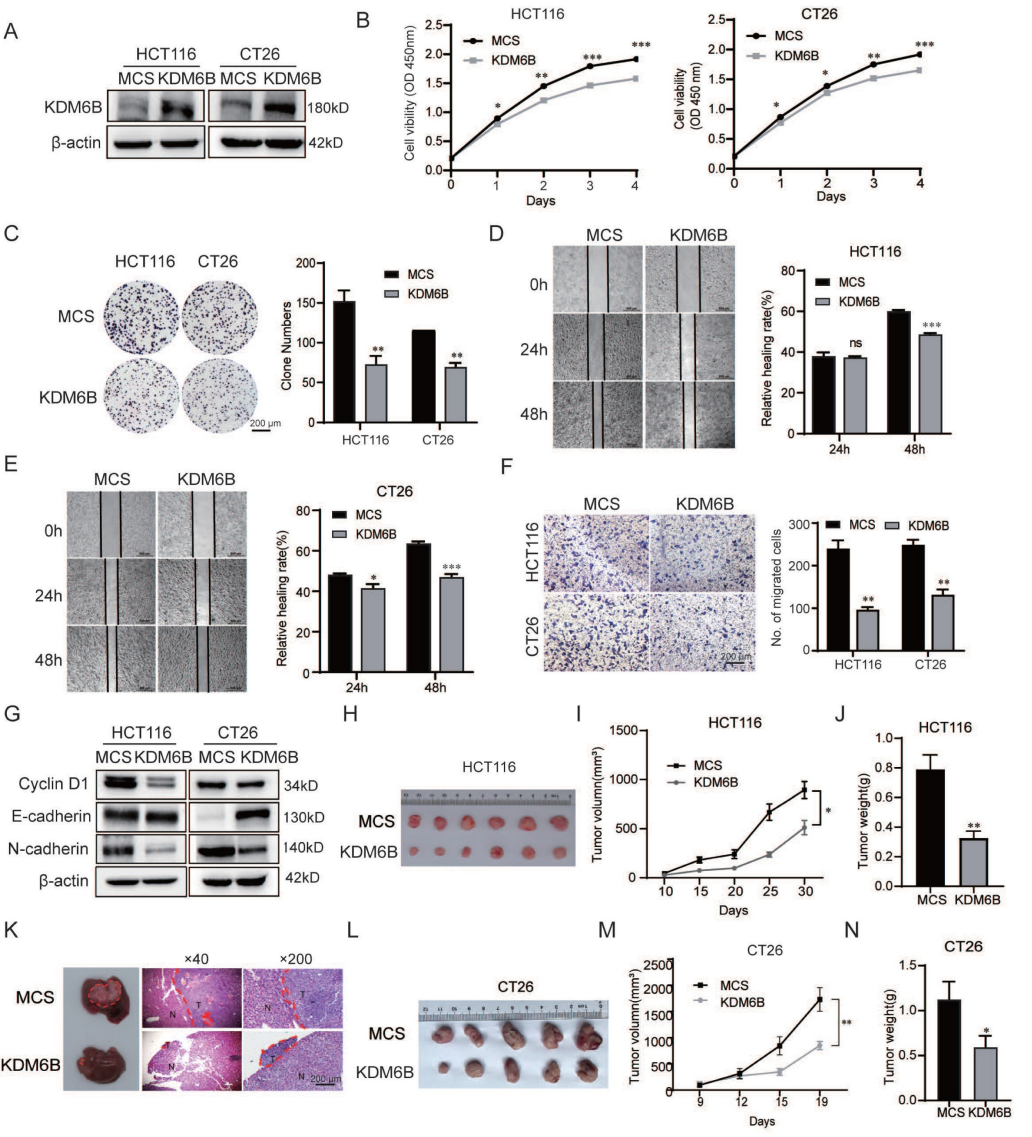
** KDM6B inhibits the malignant characteristics of CRC cells *in vitro* and* in vivo.*** Western blot analysis of KDM6B expression in HCT116 and CT26 cells overexpressing KDM6B or in empty vector (MCS) cells. **(B)** A CCK8 assay was used to analyze the viability of HCT116 and CT26 cells overexpressing KDM6B or MCS. **(C)** Representative images and statistical analysis of the plate colony formation assay results. **(D-E)** Representative images and statistical analysis of the relative healing rate was performed using a wound healing assay. **(F)** Representative images and statistical analysis of the transwell assay results. **(G)** Western blotting was used to analyze the expression of Cyclin D1, E-cadherin and N-cadherin. **(H-I)** Representative images of tumor size and statistical analysis of tumor volume in a mouse model of CRC established via implantation of HCT116 cells overexpressing KDM6B or MCS. (**J**) Statistical analysis of the tumor weight. (**K**) Liver metastasis models were established via intrasplenic injection of 3 × 10⁶ HCT116 cells overexpressing KDM6B or MCS per mouse. Metastatic nodules of the liver and representative images of HE-stained metastatic lesions. (**L-M**) Representative images of tumor size and statistical analysis of tumor volume in a mouse model of colorectal cancer established via implantation of CT26 cells overexpressing KDM6B or MCS. (**N**) The tumor weight results. The data are shown as the means ± SEMs of three independent experiments. *P < 0.05, **P < 0.01, ***P < 0.001.

To investigate the role of KDM6B in colorectal cancer cells, we constructed a stable overexpression vector for KDM6B in the human CRC cell line HCT116 and mouse CRC cell line CT26. Western blotting was performed to validate the overexpression level of KDM6B (**Fig. 2A**). A CCK8 assay was performed to evaluate the viability of CRC cells, and the results showed that KDM6B overexpression significantly reduced the viability of CRC cells (**Fig. 2B**). In addition, colony formation assay showed that the overexpression of KDM6B reduced the number of CRC cell colonies (**Fig. 2C**). Wound healing and Transwell assays further revealed that KDM6B overexpression attenuated the migration capacity of CRC cells (**Fig. 2D**-**F**). We also detected the expression of proliferation-related marker Cyclin D1 and metastasis related marker E-cadherin and N-cadherin. Our results showed that KDM6B overexpression inhibited the proliferation, metastasis and invasion ability of CRC cells (**Fig. 2G**).

We further verified the above findings *in vivo* by establishing subcutaneous tumor and liver metastasis mouse models of CRC, using KDM6B-overexpressing HCT116 or control cells for injection. The results showed that KDM6B overexpression significantly reduced the tumor volume, tumor weight and the liver metastatic area in CRC (**Fig. 2H-K**). Consistently, similar results were observed in a subcutaneous mouse model established with KDM6B-overexpressing CT26 cells (**Fig. 2L-N**). Taken together, these findings demonstrated that KDM6B suppresses the malignant phenotypes of CRC cells both *in vitro* and* in vivo*.

### KDM6B overexpression increases the infiltration of CD8^+^ T cells in CRC mice

**Figure 3:**
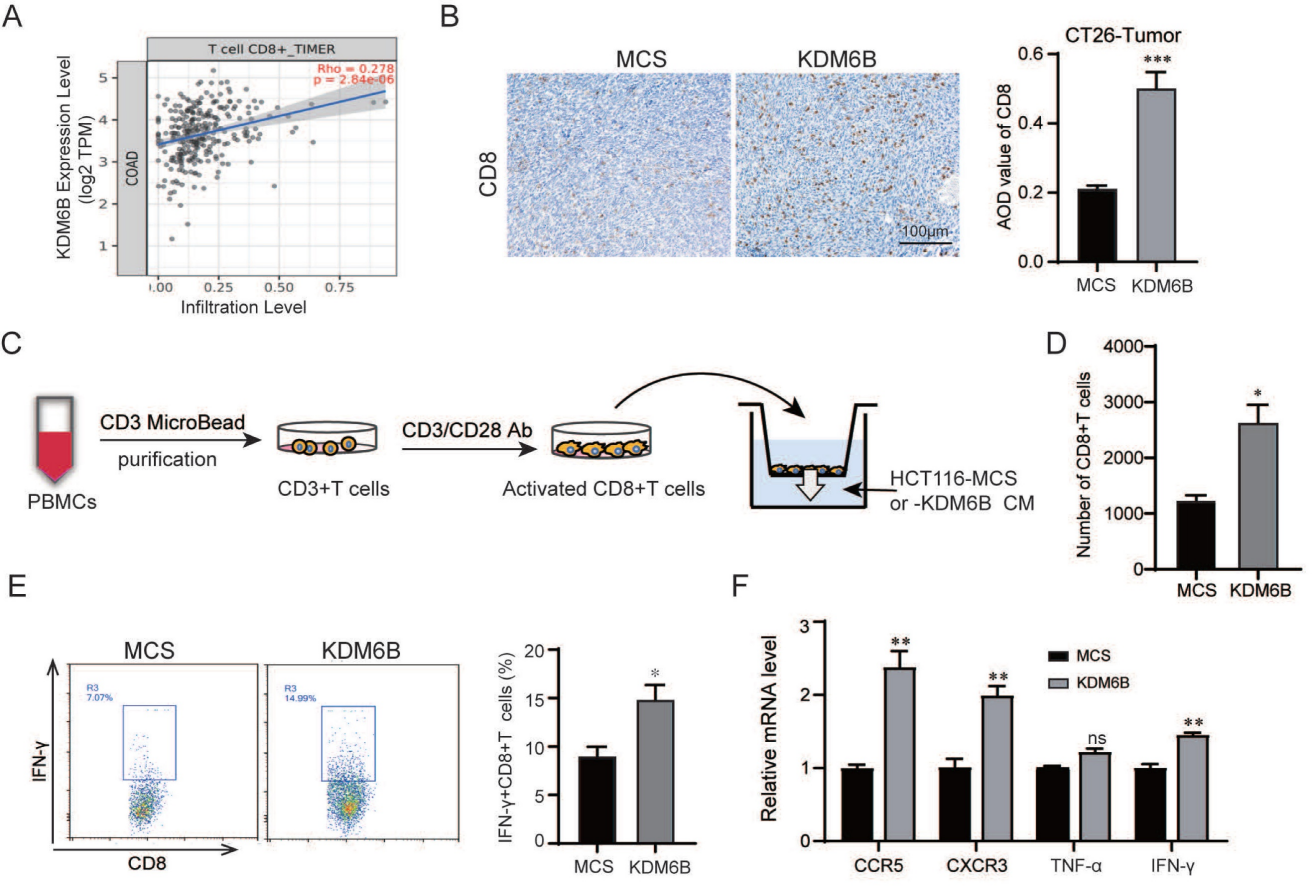
** KDM6B overexpression increases the infiltration of CD8^+^ T** c**ells in CRC mice. (A)** Correlation analysis between KDM6B and CD8^+^ T cells in CRC according to TIMER data. **(B)** IHC staining of CD8^+^ T cells. (**C**) CD3^+^ T cells purified from human peripheral blood mononuclear cells (PBMCs) were activated to CD8^+^ T cells by an anti-CD3/CD28 antibody and then stimulated with conditioned medium (CM) from HCT116 cells overexpressing KDM6B or MCS. A schematic representation was shown. (**D**) Statistical analysis of the number of chemotactic CD8^+^ T cells. (**E**) Representative flow cytometric plots and percentages of IFN-γ^+^ CD8^+^ T cells. (**F**) RT-qPCR analysis of the expression of CCR5 and CXCR3 and the effective factors TNF-α and IFN-γ in chemotactic CD8^+^ T cells. The data are shown as the means ± SEMs of three independent experiments. *P < 0.05, **P < 0.01, ***P < 0.001.

We next explored the mechanism by which KDM6B inhibits tumor progression. Previous study have focused on the role of KDM6B in CRC cells, but relatively few investigations have addressed its contribution to the immune microenvironment. As is known, immune cells, especially CD8^+^ T cytotoxic cells, play a critical role in suppressing tumor progression. This finding prompted us to investigate whether KDM6B affects the infiltration of CD8^+^ T cells into tumors. We first analyzed the correlation between the expression of KDM6B and the infiltration of CD8^+^ T cells in CRC using the TIMER database. The results showed a positive correlation between KDM6B expression and CD8^+^ T-cell infiltration (**Fig. 3A**). This finding was further confirmed by immunohistochemical staining for CD8 protein in tumor tissues (**Fig. 3B**). To investigate whether KDM6B overexpression enhances CD8^+^ T cell migration toward CRC cells, we performed *in vitro* chemotaxis assays (**Fig. 3C**). The results showed that KDM6B overexpression significantly increased the number of CD8^+^ T cells migrating toward CRC cells (**Fig. 3D**). More importantly, we examined the expression of chemokine receptors CCR5 and CXCR3, as well as IFN-γ and TNF-α, in activated CD8^+^ T cells. Flow cytometry and RT‒qPCR analysis revealed that KDM6B overexpression elevated the proportion of IFN-γ^+^ CD8^+^ T cells and upregulated the expression of CCR5, CXCR3 and IFN-γ (**Fig. 3E**-**F**). These findings indicated that KDM6B overexpression may suppress CRC progression by both promoting the infiltration and activity of CD8^+^ T cells.

### KDM6B increased the expression of PD-L1 and CD8^+^ T cells-related chemokines express in CRC cells through STAT3 signaling and demethylase activity

**Figure 4:**
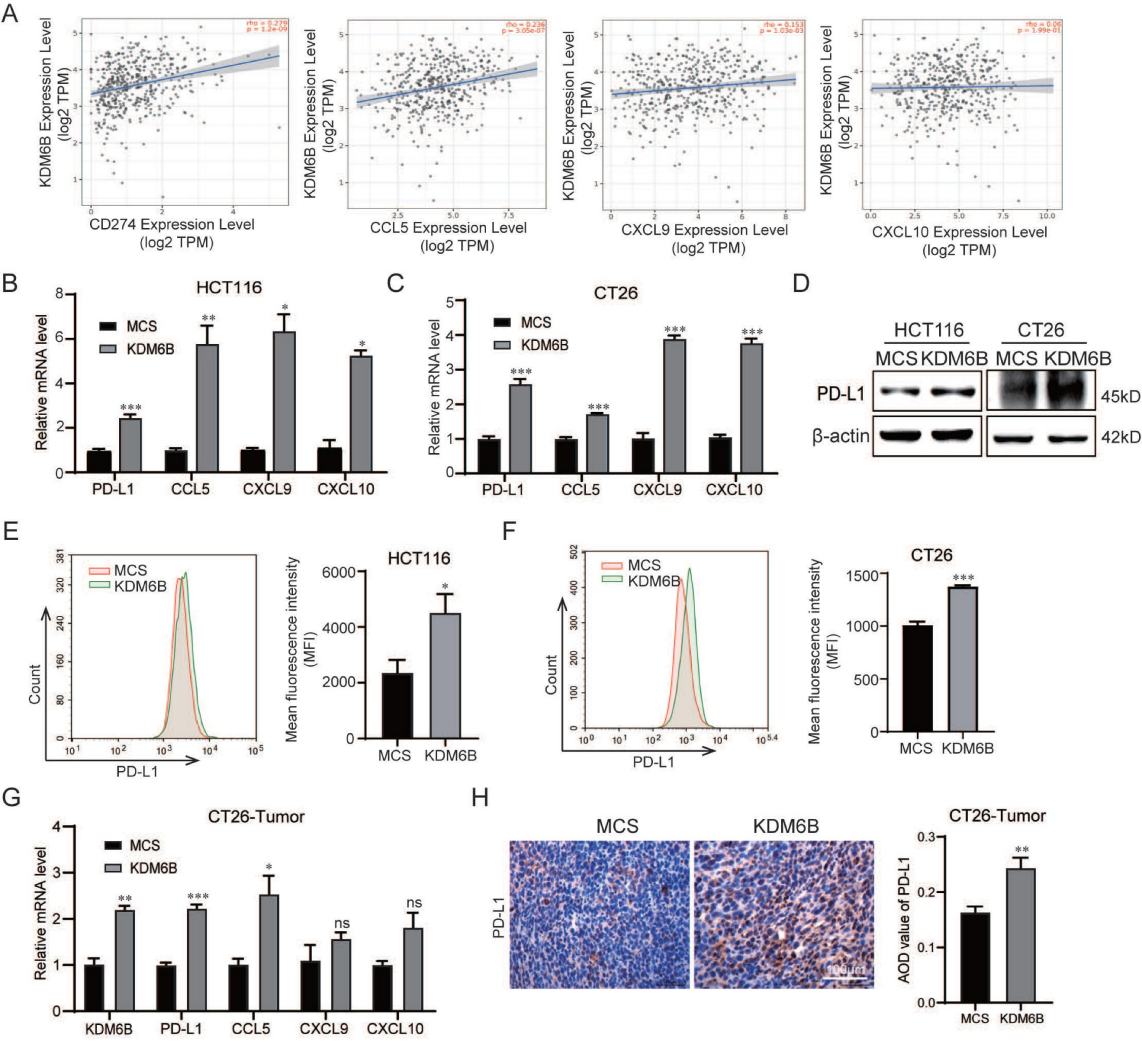
** KDM6B upregulates the expression of PD-L1 and CD8^+^ T-cell-attracting chemokines in CRC cells. (A)** Correlation analysis between KDM6B and PD-L1/CD274, as well as effector T-cell-attracting chemokines (CCL5, CXCL9, and CXCL10), based on TIMER database analysis. **(B-C)** RT-qPCR analysis of PD-L1, CCL5, CXCL9, and CXCL10 expression in HCT116 cells and CT26 cells overexpressing KDM6B and empty vector (MCS). **(D-F)** Western blot and flow cytometry analysis of PD-L1 expression in HCT116 cells and CT26 cells overexpressing KDM6B or empty vector (MCS). **(G)** RT-qPCR analysis of KDM6B, PD-L1, CCL5, CXCL9, and CXCL10 mRNA expression in tumor tissues from mice implanted with CT26 cells overexpressing KDM6B or empty vector (MCS). **(H)** IHC staining analysis of PD-L1 expression in tumor tissues. The data are shown as the means ± SEMs of three independent experiments. *P < 0.05, **P < 0.01, ***P < 0.001.

Accumulating evidence indicates that PD-L1 and the attracting chemokines CCL5, CXCL9, and CXCL10 are important factors for CD8^+^ T-cell activity and infiltration. To determine whether KDM6B modulates the expression of these molecules, we first performed a correlation analysis using the TIMER database. The results showed that a positive correlation between KDM6B expression and the expression of CCL5, CXCL9, CXCL10, and PD-L1 **(Fig. 4A)**. RT-qPCR analysis revealed that the mRNA expression levels of PD-L1, CCL5, CXCL9 and CXCL10 were significantly upregulated in KDM6B-overexpressing CRC cells compared with control cells **(Fig. 4B-C)**. Western blot analysis further confirmed higher PD-L1 protein expression in KDM6B-overexpressing cells than in control cells **(Fig. 4D)**. Flow cytometry analysis also demonstrated that KDM6B overexpression enhanced PD-L1 expression in both human CRC cells (HCT116) and murine CRC cells (CT26) **(Fig. 4E-F)**. Consistently, mRNA expression analysis of subcutaneous tumor tissues derived from CT26 cell injections showed analogous upregulation of PD-L1, CCL5, CXCL9, and CXCL10 **(Fig. 4G)**. Correspondingly, IHC staining showed that the expression of PD-L1 increased in tumor tissues overexpressing KDM6B compared to that in the control group **(Fig. 4H)**. These findings suggest that KDM6B overexpression promotes the expression of PD-L1 and CD8^+^ T-cell-attracting chemokines in CRC.

**Figure 5:**
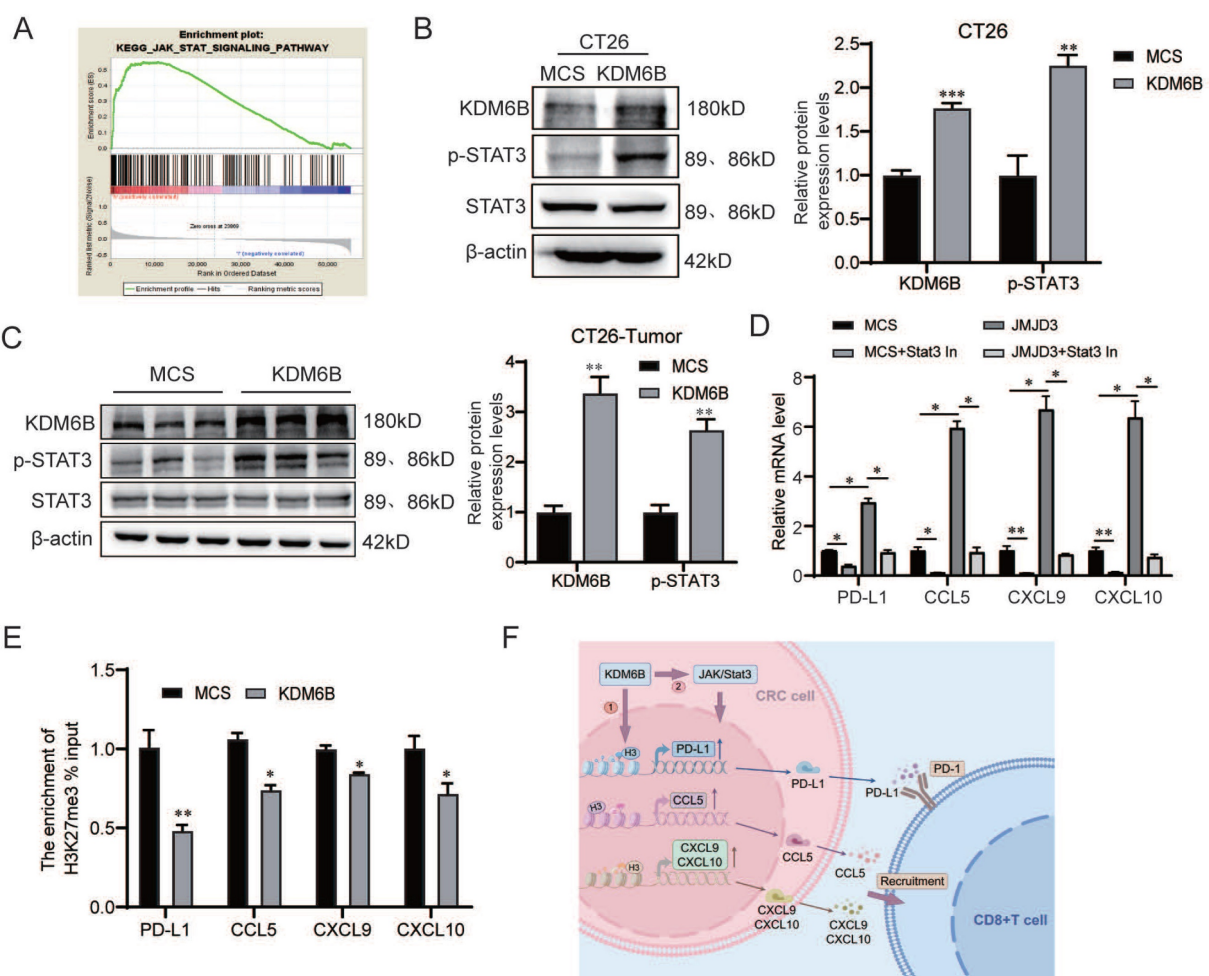
** KDM6B depends on STAT3 signaling and its demethylase activity to promote PD-L1 and CD8^+^ T-cell-attracting chemokine expression. (A)** GSEA analysis of JAK/STAT signaling pathway enrichment in CRC patients with different KDM6B expression levels. **(B)** Western blot analysis of the expression of KDM6B, p-STAT3 and STAT3, and their statistical results in CT26-overexpressing MCS and KDM6B cells. **(C)** Western blot analysis of the expression of KDM6B, p-STAT3 and STAT3 in tumor tissues from mice implanted with CT26 cells overexpressing MCS or KDM6B, with statistical analysis of the data.** (D)** HCT116 cells overexpressing KDM6B or MCS were treated with a STAT3 inhibitor (10 μM, 24 h), followed by RT-qPCR analysis of PD-L1, CCL5, CXCL9, and CXCL10 mRNA expression. **(E)** ChIP-qPCR analysis of the enrichment levels of H3K27me3 on the promoters of PD-L1, CCL5, CXCL9, and CXCL10 in HCT116 cells overexpressing KDM6B or MCS. **(F)** Schematic illustration the regulation of KDM6B-mediated regulation of CD8^+^ T cells: KDM6B in CRC cells promotes PD-L1 and CD8^+^ T-cell-attracting chemokine expression via STAT3 signaling and its demethylase activity. The data are shown as the means ± SEMs of three independent experiments. *P < 0.05, **P < 0.01, ***P < 0.001.

Next, we investigated the mechanism by which KDM6B regulates the expression of PD-L1 and CD8^+^ T cells-related chemokines. It has been reported that phosphorylated STAT3 (p-STAT3) translocates to the nucleus and binds to the promoters of these genes, enhancing their transcription^17^-^19^. We first evaluated the correlation between STAT signaling and KDM6B expression by using the KEGG database. The results showed that high level of KDM6B was associated with enrichment and increased activity of the JAK/STAT signaling pathway **(Fig. 5A)**. These findings led us to speculate that KDM6B may regulate the expression of PD-L1 and CD8^+^ T-cell-attracting chemokines via activation of the STAT3 signaling pathway. To validate this hypothesis, we detected STAT3 phosphorylation *in vitro and in vivo*. Our data showed that KDM6B overexpression significantly increased phosphorylated STAT3 levels in CRC cells and tumor tissues compared with the control group **(Fig. 5B-C)**. Furthermore, inhibition of STAT3 activity by using a STAT3 inhibitor abrogated the promoting effect of KDM6B overexpression on the expression of these genes **(Fig. 5D)**. Collectively, these findings suggest that KDM6B may activate JAK/STAT3 signaling to upregulate PD-L1, CCL5, CXCL9 and CXCL10 expression.

Given that KDM6B regulates target expression through its demethylase activity, we further investigated whether its regulatory effects on the expression of the above genes are dependent on its enzymatic activity. ChIP‒qPCR analysis revealed that overexpression of KDM6B resulted in a significant reduction in H3K27me3 enrichment in the promoter regions of the target genes (PD-L1, CCL5, CXCL9 and CXCL10) (**Fig. 5E**). Taken together, these results indicate that KDM6B upregulates the expression of PD-L1 and CD8^+^ T-cell-attracting chemokines through its demethylase activity and activation of the STAT3 signaling, thereby promoting CD8^+^ T cells infiltration in CRC (**Fig. 5F**).

### The inducer of KDM6B, paricalcitol, enhances the anti-colorectal cancer effect of anti-PD-L1 therapy

**Figure 6:**
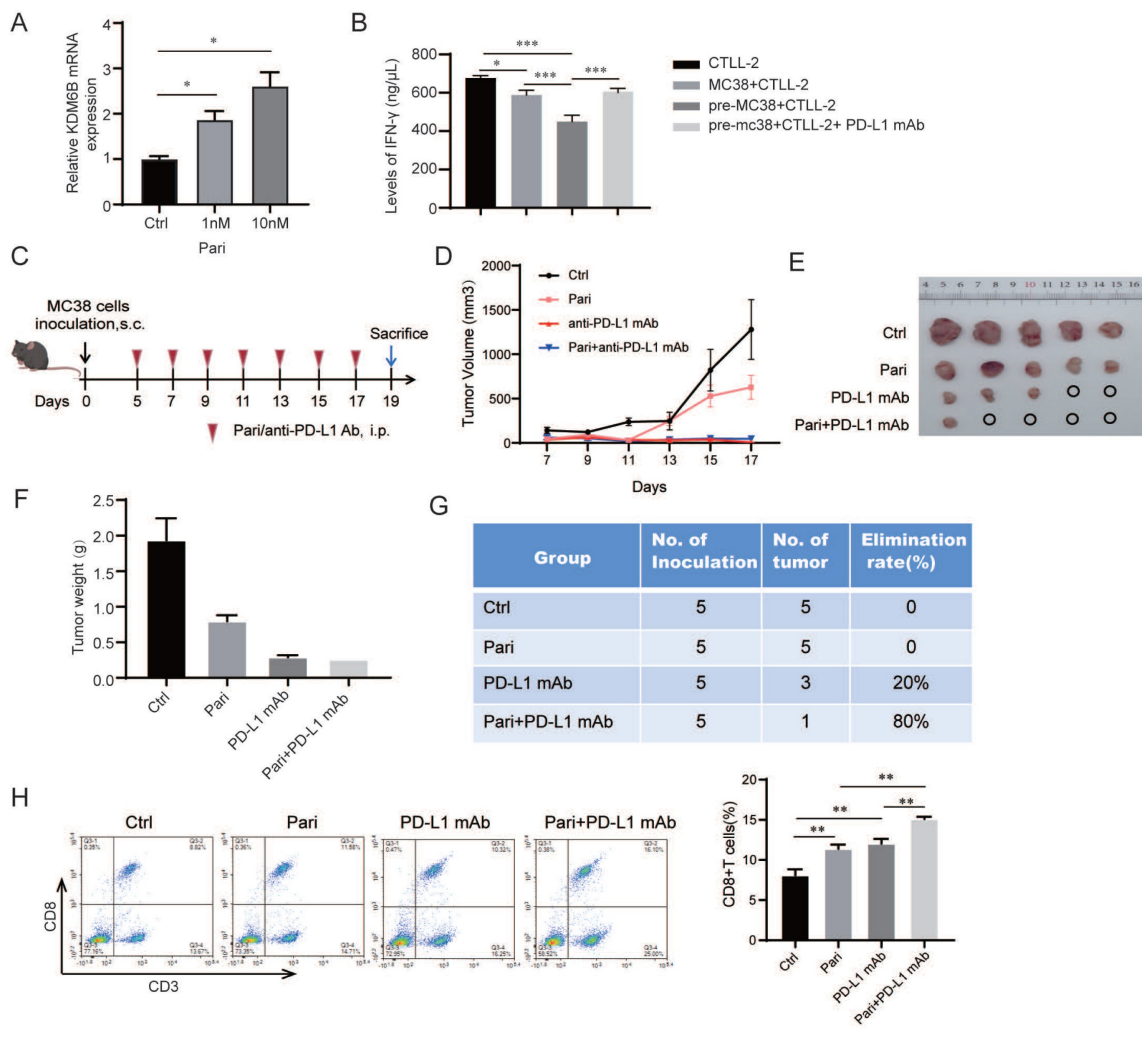
** The inducer of KDM6B paricalcitol enhances the efficacy of anti-PD-L1 antibody in CRC mice. (A)** MC38 cells were treated with paricalcitol (Pari) (1, 10 nM) for 24 h. RT-qPCR analysis of KDM6B mRNA levels were performed.** (B)** Paricalcitol (Pari)-pretreated MC38 cells were cocultured with murine CTLL-2 cytotoxic T cells at a ratio of 1:1, then treated with or without an anti-PD-L1 antibody (200 nM) for 24 h. The level of IFN-γ in the supernatant was measured by ELISA. **(C)** A CRC mouse model was established via subcutaneous injection of MC38 cells, followed by intraperitoneal injection of paricalcitol (Pari), anti-PD-L1 antibody, or their combination. A schematic of the experimental design is shown. **(D)** Analysis of tumor volume. **(E)** Representative images of subcutaneous tumors. **(F)** Statistical analysis of the tumor weight. **(G)** Statistical analysis of the tumor elimination rate. **(H)** Flow cytometric analysis of CD8^+^ T cells proportion in mouse peripheral blood. The data are shown as the means ± SEMs of three independent experiments. *P < 0.05, **P < 0.01, ***P < 0.001.

The above results suggest that upregulation of KDM6B expression enhances CD8^+^ T-cell infiltration in colorectal tumor tissues and potentiates the therapeutic efficacy of immune checkpoint inhibitors. Additionally, previous studies, including ours, have demonstrated that the vitamin D receptor agonist paricalcitol can induce KDM6B upregulation^11^, ^14^, which was also confirmed in CRC cells (**Fig. 6A**). We then cocultured the paricalcitol-pretreated mouse CRC cell line MC38 with the murine cytotoxic T-cell line CTLL-2 at a ratio of 1:1, and treated the cells with an anti-PD-L1 antibody. The results showed that the combination of the anti-PD-L1 antibody significantly increased the secretion level of IFN-γ in the supernatant compared with paricalcitol monotherapy (**Fig. 6B**). To validate these findings *in vivo*, we established a subcutaneous CRC mouse model using MC38 cells to evaluate the antitumor effect of paricalcitol combined with an anti-PD-L1 antibody **(Fig. 6C)**. The results showed that treatment with either paricalcitol or the PD-L1 antibody alone reduced tumor growth, with the anti-PD-L1 antibody demonstrating a more pronounced effect. Although tumor size did not differ significantly between the anti-PD-L1 antibody group and the combination treatment group, the combined therapy exhibited superior tumor elimination **(Fig. 6D-G)**. Flow cytometry analysis of peripheral blood CD8^+^ T-cell proportions further showed that the combination of paricalcitol and anti-PD-L1 antibody significantly increased the proportion of CD8^+^ T cells in mice **(Fig. 6H)**. Taken together, these findings indicate that paricalcitol, a KDM6B inducer, enhances the antitumor efficacy of anti-PD-L1 antibody therapy.

## 4. Discussion

Due to its high morbidity and mortality, colorectal cancer is considered a global health issue, and new therapeutic are urgently needed^20^,^21^. In colorectal cancer, T-cell infiltration to the tumor site is associated with a favorable prognosis, suggesting a possible role for immune regulation in suppressing tumor growth. Immunotherapy aims to harness the immune system to fight cancer^22^-^24^. Previous studies have shown that the H3K27 histone demethylase KDM6B plays a dual role in immune diseases. Furthermore, the role of KDM6B in cancer is complex and controversial^25^,^26^. In addition, immunotherapy is a hot topic in current tumor research. Improving the sensitivity of CRC to immunotherapy and enhancing antitumor immunity are important clinical issues. Therefore, the aim of this study was to investigate the role of KDM6B in CRC progression and its regulation of tumor immunity.

In this study, we found that KDM6B expression is downregulated in tumor tissues and cell lines. KDM6B overexpression suppressed the proliferation and metastasis of colorectal cancer cells in vivo and in vitro, which was consistent with its role in neuroblastoma^26^. In addition, KDM6B expression correlated with the prognosis of surgically resected CRC patients and was much greater in normal tissues than in CRC tissues^14^. These findings suggested that KDM6B plays a suppressive role in CRC progression.

Notably, we found that KDM6B overexpression did not significantly alter E-cadherin expression in HCT116 cells or Cyclin D1 expression in CT26 cells. The differences in protein changes between the two cell lines suggest that the mechanism of KDM6B action may vary across different cell types. On one hand, cell type-specific and metabolic differences. HCT116 (human-derived) and CT26 (mouse-derived) colon cancer cells exhibit distinct genomic backgrounds, which may involve different mutational profiles that influence KDM6B's regulatory mechanisms; KDM6B activity is sensitive to intracellular metabolic modifications^27^, and metabolic variations between the two cells may affect KDM6B-mediated gene regulation. On the other hand, different histone modification states at the promoters of E-cadherin and Cyclin D1 genes between the cell lines may underlie their differential responses to KDM6B. Furthermore, other epigenetic regulators may contribute to this divergence. For instance, EZH2, a core component of the polycomb repressive complex 2 (PRC2), antagonizes KDM6B by catalyzing H3K27 di-/tri-methylation to suppress gene expression^28^. This interplay may explain the observed minimal changes in E-cadherin and Cyclin D1 expression. In addition, complexity of signaling networks. Research indicates that E-cadherin expression is suppressed by transcriptional repressors including Snail, Twist, and ZEB1/ZEB2. Conversely, miR-200b and miR-200c positively regulate E-cadherin through targeted degradation of its repressors' (ZEB1/ZEB2) transcripts. Additionally, miRNA-9 negatively modulates E-cadherin expression by degrading its mRNA transcripts^29^. Similarly, Cyclin D1 expression is cross-regulated by multiple signaling pathways, such as MAPK/ERK, PI3K/AKT/mTOR, Wnt/β-catenin, NF-κB, FBX4, etc^30^. These pathways collectively influence Cyclin D1 levels through transcriptional regulation, translational control, and protein stability modulation. Therefore, the complex signal network may lead to the insignificant effect of KDM6B on the expression of E-cadherin and Cyclin D1.

It is known that CD8^+^ T cells in the tumor immune microenvironment play a key role in antitumor effects and are the backbone of tumor immunotherapy. In our present study, we also found that overexpression of KDM6B promoted the infiltration of CD8^+^ T cells into CRC tissues and increased the expression of PD-L1 and CD8^+^ T-cell -attracting chemokines (CCL5, CXCL9, and CXCL10). Notably, high PD-L1 expression is a hallmark of “hot” tumors and predicts actionable immune activation, although not all patients who receive PD-L1/PD-1 targeted therapy have good treatment efficacy, and only 20-40% of patients benefit from these new therapies^31^,^32^. Adaptive PD-L1 expression indicates increased response rates to immunotherapy and chemotherapy. Therefore, it suggests that KDM6B overexpression may enhance the sensitivity of CRC to PD-1/PD-L1 antibody therapy by increasing PD-L1 expression.

Finally, it was considered that MC38 tumors with “hot” tumor microenvironment and sensitive to immunotherapy compared to CT26 “cold” tumor^33^,^34^. We therefore selected the more immunoreactive MC38 cells to investigate the combined anti-CRC effect of KDM6B inducer paricalcitol and anti-PD-L1 antibody. Our data showed that paricalcitol enhanced the antitumor efficacy of anti-PD-L1 antibodies in CRC. This finding has important implications for the treatment of CRC patients. Inducing KDM6B offers several potential therapeutic advantages for CRC. Firstly, by enhancing the effectiveness of anti-PD-L1 antibodies, it provides a new strategy for immunotherapy in CRC. Immunotherapy has shown great promise in cancer treatment, but its efficacy in CRC is still limited. The ability of KDM6B inducer to boost the effect of anti-PD-L1 antibodies can help more CRC patients benefit from immunotherapy, potentially increasing the response rate and extending survival time. Secondly, modulating KDM6B may help overcome tumor immune evasion. Evasion of anti-tumor immunity is an important mechanism leading to treatment resistance in CRC. Inducing KDM6B with paricalcitol may disrupt these evasion mechanisms, making the tumors more vulnerable to the immune attack mediated by anti-PD-L1 antibodies. This could potentially reverse the immune- suppressive tumor microenvironment in CRC, creating a more favorable condition for the immune system to combat cancer cells.

More importantly, paricalcitol is a FDA-approved drug for secondary hyperparathyroidism in chronic kidney disease, making its repurposing for CRC immunotherapy represents a promising treatment strategy. A previous study in patients with metastatic pancreatic ductal adenocarcinoma (PDAC) receiving maintenance therapy found that paricalcitol did not enhance the clinical efficacy of pembrolizumab, likely attributable to the short half-life of the paricalcitol formulation used. However, CE-CT imaging revealed distinct tumor microenvironment alterations in the pembrolizumab-paricalcitol cohort compared to the pembrolizumab-placebo group^35^*.* Currently, several clinical trials involving paricalcitol are ongoing: a pilot study is evaluating paricalcitol in combination with standard chemo-radiation for resectable rectal cancer (NCT01197664); a phase I trial is investigating the safety and optimal dosage of paricalcitol in combination with gemcitabine in patients with advanced cancer (NCT00217477); a phase Ib pharmacodynamic trial (NCT03300921) is assessing paricalcitol as neoadjuvant therapy for resectable pancreatic cancer. However, clinical trials on paricalcitol's role in CRC immunotherapy have not been carried out. All these studies will critically inform future research of paricalcitol as a treatment for CRC. In conclusion, inducing KDM6B with paricalcitol represents a novel and potentially effective approach to improve the treatment outcomes of CRC patients, offering new hope for their therapeutic management.

## 5. Conclusion

In conclusion, our study revealed that KDM6B overexpression inhibited the progression of CRC and promoted the infiltration of CD8^+^ T cells. Mechanistically, KDM6B promoted CD8^+^ T-cell infiltration by upregulating PD-L1 and the CD8^+^ T-cell-attracting chemokines CCL5, CXCL9, and CXCL10 expression, via its demethylase activity and activation of the STAT3 signaling pathway. Furthermore, we demonstrated that a KDM6B inducer paricalcitol potentiated the antitumor efficacy of the PD-L1 antibody against CRC. The study provides a theoretical foundation and a potential strategy for enhancing the efficacy of CRC immunotherapy.

## Figures and Tables

**Table 1:** primer sequences for RT-qPCR

Gene	Forward (5'-3')	Reverse (5'-3')
mus-GAPDH	CATCACTGCCACCCAGAAGACTG	ATGCCAGTGAGCTTCCCGTTCAG
mus-KDM6B	AGACCTCACCATCAGCCACTGT	TCTTGGGTTTCACAGACTGGGC
mus-PD-L1	TGCGGACTACAAGCGAATCACG	CTCAGCTTCTGGATAACCCTCG
mus-CCL5	CCTGCTGCTTTGCCTACCTCTC	ACACACTTGGCGGTTCCTTCGA
mus-CXCL9	CCTAGTGATAAGGAATGCACGATG	CTAGGCAGGTTTGATCTCCGTTC
mus-CXCL10	ATCATCCCTGCGAGCCTATCCT	GACCTTTTTTGGCTAAACGCTTTC
Hu-GAPDH	GTCTCCTCTGACTTCAACAGCG	ACCACCCTGTTGCTGTAGCCAA
Hu-KDM6B	GACCCTCGAAATCCCATCACAG	GTGCGAACTTCCACGGTGTGTT
Hu-PD-L1	TGCAGGGCATTCCAGAAAGA	TAGGTCCTTGGGAACCGTGA
Hu-CCL5	CAGTCGTCTTTGTCACCCGA	CGGGTGGGGTAGGATAGTGA
Hu-CXCL9	TGAGAAAGGGTCGCTGTTCC	GGGCTTGGGGCAAATTGTTT
Hu-CXCL10	AGCAGAGGAACCTCCAGTCT	ATGCAGGTACAGCGTACAGT
Hu-PD1	CAGTTCCAAACCCTGGTGGT	GGCTCCTATTGTCCCTCGTG
Hu-CCR5	TCTCTTCTGGGCTCCCTACAAC	CCAAGAGTCTCTGTCACCTGCA
Hu-CXCR3	ACGAGAGTGACTCGTGCTGTAC	GCAGAAAGAGGAGGCTGTAGAG
Hu-TNFα	CTCTTCTGCCTGCTGCACTTTG	ATGGGCTACAGGCTTGTCACTC
Hu-IFN γ	GAGTGTGGAGACCATCAAGGAAG	TGCTTTGCGTTGGACATTCAAGTC

**Table 2:** Primer sequences for ChIP-qPCR

Gene	Forward (5'-3')	Reverse (5'-3')
Hu-PD-L1	AAGGAAAGGCAAACAACG	TCCGCCAAAGTGCTGATA
Hu-CXCL9	ATGTCCTTAGCCCACTTT	AACAGACAACCCACAGAG
Hu-CXCL10	AACCTGTTTCCCTTCTGT	CATTTGGGTATCTGATTTGT
Hu-CCL5	AGGGCAACTGGGTTCTGA	ACCGAGGGCTTATCTGTG

## Abbreviations

CRC: colorectal cancer
KDM6B: lysine-specific demethylase 6B
IHC: immunohistochemistry
RT-qPCR: real-time quantitative PCR
ELISA: enzyme-linked immunosorbent assay
IFN-γ: interferon γ
STAT3: signal transducer and activator of transcription 3
PD-L1: programmed cell death 1 ligand 1
CCL5: C-C motif chemokine ligand 5
CXCL9: CXC-chemokine ligand 9
CXCL10: CXC-chemokine ligand 10
MCS: empty vector
